# Preferred prenatal counselling at the limits of viability: a survey among Dutch perinatal professionals

**DOI:** 10.1186/s12884-017-1644-6

**Published:** 2018-01-03

**Authors:** Ms Rosa Geurtzen, Arno Van Heijst, Rosella Hermens, Hubertina Scheepers, Mallory Woiski, Jos Draaisma, Marije Hogeveen

**Affiliations:** 1 Amalia Children’s Hospital, Department of Pediatrics, Radboud university Medical Center, PO Box 9101, 6500HB Nijmegen, The Netherlands; 2 Scientific Institute for Quality of Care, Radboud university medical center, Nijmegen, The Netherlands; 3 Department of Gynecology, Maastricht UMC+, Maastricht, The Netherlands; 4 Amalia Children’s Hospital, Department of Gynecology, Radboud University Medical Center, Nijmegen, The Netherlands

**Keywords:** Counselling, Decision-making, (extreme) prematurity, (limits of) viability

## Abstract

**Background:**

Since 2010, intensive care can be offered in the Netherlands at 24^+0^ weeks gestation (with parental consent) but the Dutch guideline lacks recommendations on organization, content and preferred decision-making of the counselling. Our aim is to explore preferred prenatal counselling at the limits of viability by Dutch perinatal professionals and compare this to current care.

**Methods:**

Online nationwide survey as part of the PreCo study (2013) amongst obstetricians and neonatologists in all Dutch level III perinatal care centers (*n* = 205).The survey regarded prenatal counselling at the limits of viability and focused on the domains of organization, content and decision-making in both current and preferred practice.

**Results:**

One hundred twenty-two surveys were returned out of 205 eligible professionals (response rate 60%). Organization-wise: more than 80% of all professionals preferred (but currently missed) having protocols for several aspects of counselling, joint counselling by both neonatologist and obstetrician, and the use of supportive materials. Most professionals preferred using national or local data (70%) on outcome statistics for the counselling content, in contrast to the international statistics currently used (74%). Current decisions on initiation care were mostly made together (in 99% parents *and* doctor). This shared decision model was preferred by 95% of the professionals.

**Conclusions:**

Dutch perinatal professionals would prefer more protocolized counselling, joint counselling, supportive material and local outcome statistics. Further studies on both barriers to perform adequate counselling, as well as on Dutch outcome statistics and parents’ opinions are needed in order to develop a national framework.

**Trial registration:**

Clinicaltrials.gov, NCT02782650, retrospectively registered May 2016.

**Electronic supplementary material:**

The online version of this article (10.1186/s12884-017-1644-6) contains supplementary material, which is available to authorized users.

## Background

The anticipated delivery of an extremely premature infant at the limits of viability confronts parents as well as perinatal professionals with medical, ethical and emotional issues; especially when a decision on the initiation of care has to be made. Since the first publication in 2002 by the American Academy of Pediatrics several (albeit different) guidelines and, recommendations and comments on periviability counselling have been published [1–13]. However, there is no universally accepted way of performing prenatal counselling and, consequently, studies describe heterogeneous counselling practices worldwide [14–25].

Some guidelines on resuscitation at the limits of viability have included recommendations on the parental involvement in the decision-making process. However, both the extent of involvement of parents, as well as the range of gestational ages (GA) at which parents should be involved, varies between countries [8, 9, 11, 26].

In 2010, the Dutch guideline on perinatal practice in extremely premature delivery lowered the limit offering intensive care from 25^+0^ to 24^+0^ weeks GA. Just as some international guidelines which include a role for parents the Dutch guideline explicitly requires informed consent of parents when initiating intensive care at 24 weeks GA [27]. Although this guideline acknowledges the importance of prenatal counselling, recommendations on organization, content or decision-making of the counselling are very limited. A pilot-study exploring prenatal counselling in a simulated setting in a Dutch and American cohort (2010), showed heterogeneity in content and decision-making [28]. Although there are some recommendations on counselling [1–11], they may not be generally applicable in the Netherlands since cross-cultural differences in perinatal practices, healthcare organization, and physician and patient views are likely to exist [8, 9, 11, 26–31].

To compose a national framework on prenatal counselling at the limits of viability (currently 24 weeks GA in the Netherlands), the nationwide PreCo study (Prenatal Counselling in Prematurity) was designed, examining both professional and parental views. High quality of care originates when no differences exist between preferred and current counselling with uniformity between the involved caregivers (obstetricians and neonatologists) and specified to the needs of those receiving counselling [17, 21, 22].

The views of parents are at least as important as the view of the professionals in the topic of prenatal counselling at the limits of viability, and they will be studied separately. The primary aim of this study is to explore preferences amongst Dutch perinatal professionals on prenatal counselling at the limits of viability on three domains: *organization*, *content,* and *decision-making-process.* The secondary aim is to study differences between preferred and current counselling and between counselling preferences of neonatal and obstetrical professionals.

## Methods

### Study design

Cross-sectional study (PreCo survey) using an online survey.

### Setting and study population

This study is part of the PreCo study, evaluating Dutch care in (imminent) extremely preterm birth including current and preferred counselling, barriers and facilitators for preferred counselling from both obstetrician and neonatologist, as well as parents’ views on this (clinicaltrials.gov, NCT02782650 & NCT02782637). The results of the studies in parents are described [32] and will be described separately.

The care for extreme preterm births is centralized in the Netherlands in 10 level III centers for perinatal care which all participated in this study. Surveys were sent to all fellows and senior staff members in both obstetrics and neonatology. Data were collected from July 2012 through October 2013, approximately two to 3 years after the introduction of the new Dutch guideline on perinatal practice in extreme premature delivery.

### Survey design and data collection

We developed the current survey in three stages just as described elsewhere. The first version was based on a combination of literature on prenatal counselling, several prenatal counselling surveys that were kindly shared with us [5, 16, 17, 33–35], observations from previous Dutch studies [28], and on public discussions generated by the Dutch guideline on perinatal practice in extreme premature delivery [27]. This survey was improved in two Delphi rounds containing both four team members and two independent professionals. The entire PreCo-survey required ~20 min to complete. The survey was adapted for both professional groups to exclude irrelevant questions and to optimize the participation rate.

The content of the PreCo survey included two topics on the care for children born at the limits of viability: prenatal counselling (preferred and current) and treatment decisions [36]. For this substudy we were interested in the first: both preferred and current prenatal counselling. We defined three domains of interest to investigate this: 1) *organi*za*tion* 2) *content* and 3) the *decision-making-process.* We used a fictitious case of an ‘uncomplicated’ extreme premature delivery at 24 weeks to examine the three domains (textbox). The survey questions were designed to ask for both the preferred and current practice (Additional files 1 and 2).

**Table:** 

*Characteristics of the fictitious case*	
A consultation for prenatal counselling with an impending extreme premature delivery, singleton fetus, unremarkable history of pregnancy, average estimated fetal birth weight, unknown gender, no known congenital abnormalities, unremarkable social and medical history of parents, antenatal corticosteroids have been administered and normal fetal heart rate recording.	

An individual link to the online survey was sent to all participants. Three reminders were sent to non-responders. Survey results were anonymized before analysis. This study was exempt from IRB approval.

### Data analysis

Summary statistics were given as proportions of the respondents for that specific question. To compare preferred counselling with current counselling McNemars Ӽ^2^, Bowker McNemars Ӽ^2^ or Wilcoxon-signed-rank test were used when applicable. For comparison of the counselling methods of obstetricians and neonatologists Ӽ^2^, Fisher exact test (F.ex) or Mann Whitney U test (MWU) were used when applicable. Exact *p* values were provided, values <0.05 were considered significant. Statistical analyses were conducted using IBM SPSS Statistics (Version 20.0. Armonk, NY: IBM Corp).

## Results

### Demographics

We received 122 surveys from 205 eligible perinatal professionals^1^; a response rate of 60%. Of those, 45 were from obstetricians and 77 from neonatologists. Each Dutch perinatal center was represented by at least five respondents. Of all 122 returned surveys, eight were partially completed. Obstetricians had fewer years of experience than neonatologists (Table 1).

**Table 1:** Characteristics of perinatal professionals

	Obstetricians *(n = 84 sent)*	Neonatologists *(n = 121 sent)*
Response rate	54%	64%
Gender, *% male*	32%	69%
Having children (parent)	91%	83%
*Of those: parent of premature child (<27 weeks)*	*0%*	*2%*
Median age in years *(q25-75)*	40 (38-47)	45 (37-50)
Median years of experience *(q25-75)*	5 (1-10)	9 (4-17)*

*p 0.02 (MWU)

#### Organization of prenatal counselling

With respect to the person who should conduct the counselling of the prospective parents, perinatal professionals (91%) preferred this done by the obstetrician and neonatologist jointly, but it occurred in 61% of current practice (Table 2).

**Table 2:** Person(s) who generally conduct(s) the prenatal counselling with the parents

	Preferred	Current
Neonatologist	3%	22%
Gynecologist	0%	1%
Obstetrician + neonatologist jointly	91%	61%*
Obstetrician + neonatologist not jointly	3%	15%
Other	3%	2%

* *p* 0.01 (McNemar Bowker)

Perinatal professionals would preferably like a protocol on several aspects of prenatal counselling (Table 3); who should be counselling (94%) and at which GA (98%), which topics should be discussed (85%), and the GA at which intensive care can be offered (98%) and comfort care accepted (84%). In current practice, some of these aspects were already put into protocols.

**Table 3:** Existence of protocols for the different aspects of prenatal counselling mentioned

	*% of perinatal professionals that do have a protocol*	
	Preferred	Current
The GA at which the obstetrician or gynecologist has to ask a neonatologist or pediatrician to provide prenatal counselling to the parents	98%	80% *
The professional who conducts the consultation with the parents	94%	76% *
The topics that should at least be discussed during prenatal counselling	85%	41% *
The minimal GA for offering intensive treatment at birth	98%	88% **
The GA (upper/lower limit) at which the parents’ opinion can be decisive in whether or not to initiate intensive treatment at birth	84%	60% *

* *p* < 0.01 ** *p* = <0.05 (McNemar) comparing preferred and current practices

Neonatologists wanted to use more supporting material in their consultation (*p* < 0.01); either written (93%) or online (65%) information or a decision-aid (DA) (42%). This was different from the current situation where only 38% of the neonatologists used written information. Other modalities were used less (website 7%, video 3%, DA 1%, other 7%).

**Fig. 1:**
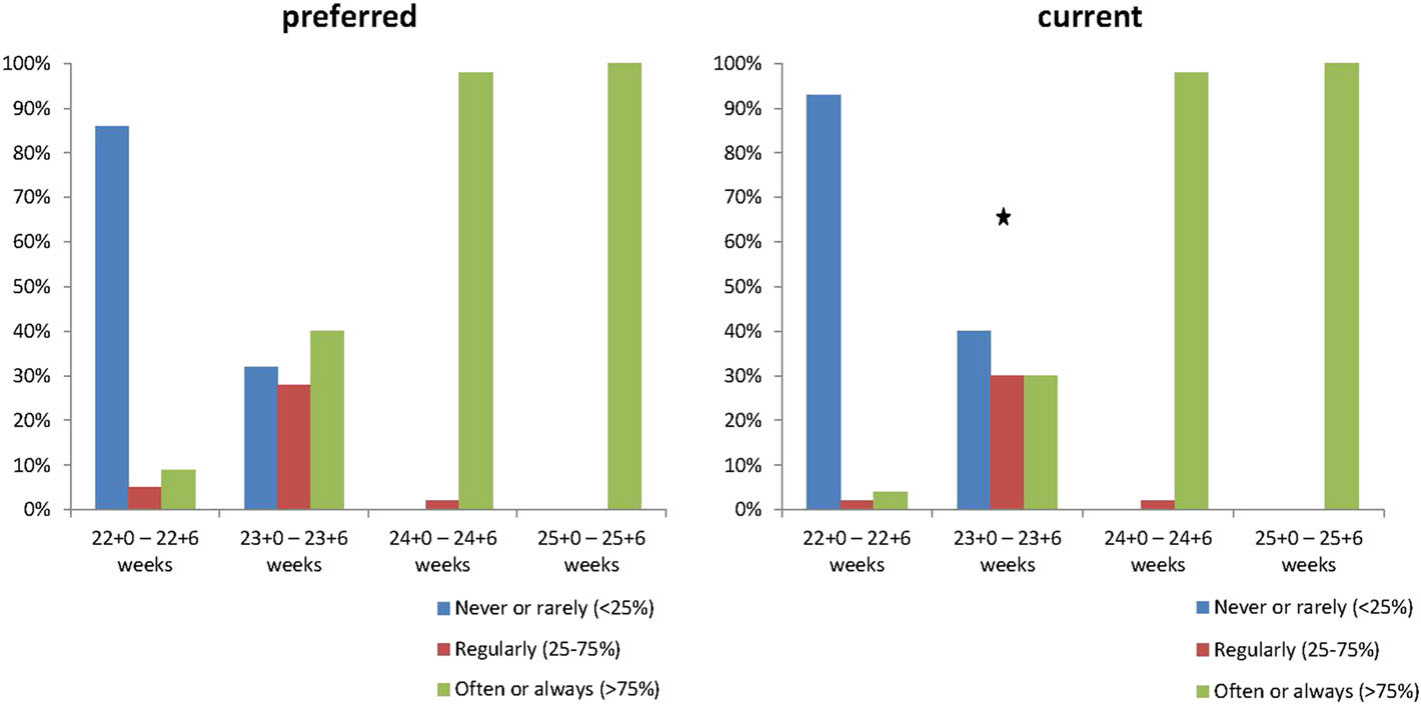
Percentages of obstetricians that ask the neonatologist for prenatal counselling in threatened preterm delivery

Starting at 24^+0/7^ weeks of GA, obstetricians preferred to ask the neonatologist often or always (98%) to provide counselling to parents in imminent preterm delivery (Fig. 1). At 22 weeks of GA, neonatologists should never or rarely be asked according to 86% of the obstetricians. At 23 weeks of GA, there was no consensus.

Of the neonatologists, 58% preferred to have more than one prenatal counselling meeting with the parents, significantly different from current practice (only 18% had more than one meeting) (*p* < 0.01). Preferably counselling should take between 15 and 45 min, comparable with current practice. The content of the consultation should be documented in both the mother’s and the infant’s medical record (76%) which was different from the current situation where it was documented only in the mother’s file (58%) (*p* < 0.01).

#### Content of prenatal counselling

An overview of topics (from a predefined list) that neonatologists think should be discussed during prenatal counselling is given in order of frequency in Table 4. The most important topics were: mortality, morbidity, intubation/ventilation and intraventricular hemorrhage.

**Table 4:** Topics preferably addressed during prenatal counselling

Topics to be discussed (preferred)	*(% of neonatologists)*
The chance the baby will have disabilities (morbidity)	96%
The chance the baby will die (mortality)	94%
Intubation and/or ventilation	93%
Intraventricular hemorrhage	91%
Cognitive impairment (e.g. mental retardation)	90%
Motor impairment (e.g. cerebral palsy)	88%
Susceptibility for (nosocomial) infections	85%
Who will be present during the delivery	82%
RDS and/or surfactant administration	78%
Expected duration of the hospital stay	75%
Breast milk and/or pumping	74%
Total Parental Nutrition (TPN)	70%
Long term pulmonary impairment	67% (*)
Non-invasive respiratory support	60%
Vision problems and/or ROP	58% (*)
Tube feeding	58%
Necrotizing enterocolitis	54%
Infection as a cause of premature delivery	49%
Social services that are available	47%
Hearing problems	47% (*)
Apneas and/or caffeine	25%
Visiting hours	17%
Hygienic rules	13% (*)
Financial consequences for the family	11% (*)

(*) *p* < 0.05 compared to current practice

**Fig. 2:**
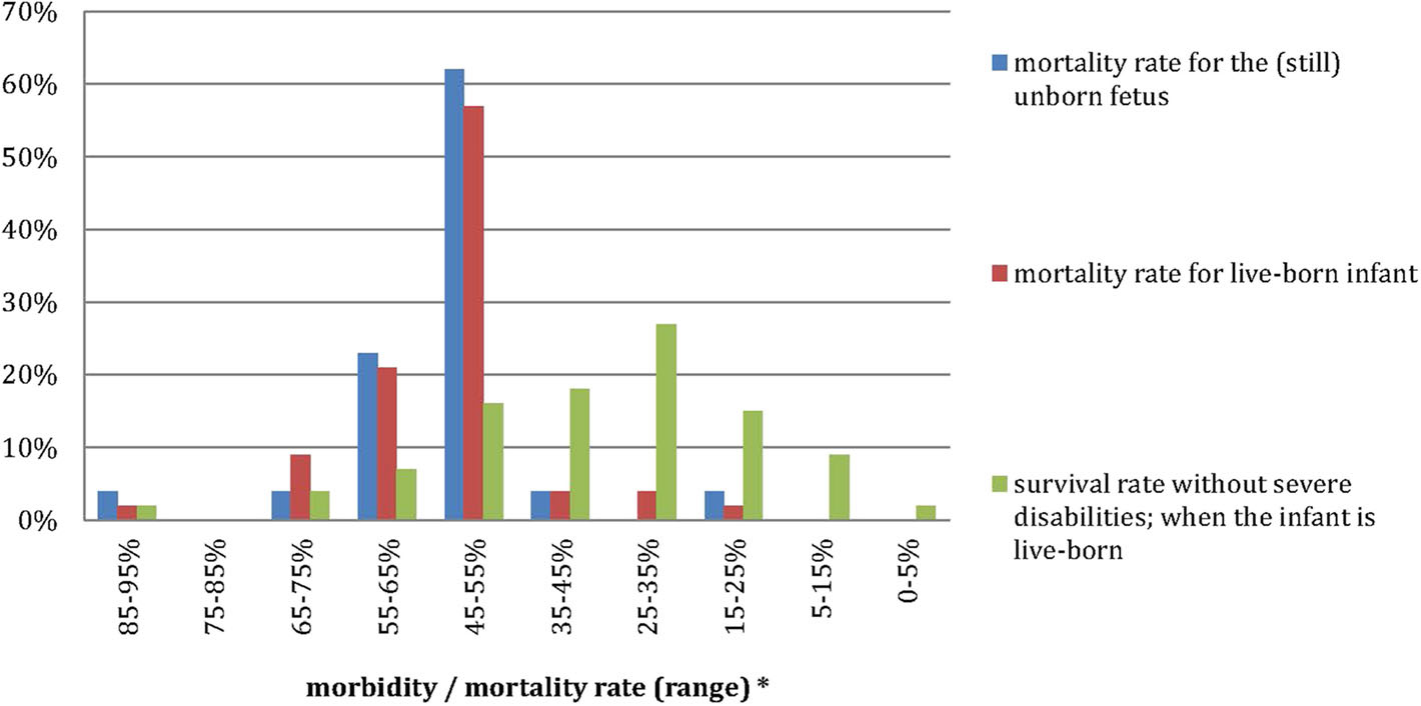
Morbidity and mortality rates currently provided by neonatologist during prenatal counselling (24 weeks GA)

When providing outcome statistics, perinatal professionals preferred to use national (48%) or hospital-specific (22%) outcome statistics. Only 21% preferred international data, which was used by the majority in current practice (74%) (p < 0.01). Not every neonatologist did provide outcome statistics in current practice: the ‘mortality rate for the unborn fetus’ was provided by 38%, the ‘mortality rate for live-born infants’ was provided by 66% and the ‘survival rate without severe disabilities’ was provided by 76%. When providing prognostic statistics, there was a wide range in the used percentages by neonatologists (Fig. 2).

#### Decision-making in prenatal counselling

**Fig. 3:**
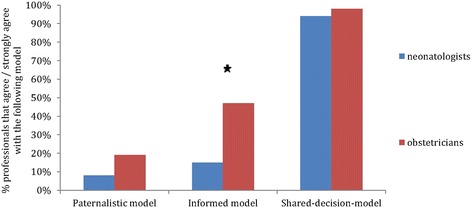
Preferred decision-making-model at 24 weeks GA on inititating intensive treatment at birth or not. Answer options: •The decision to initiate intensive treatment at birth should only be made by a health care professional (paternalistic model). •The decision to initiate intensive treatment at birth should be made by the parents, after prenatal counselling (informed model). •The decision to initiate intensive treatment at birth should be made by the health care professional and parents together (shared-decision model)

The decision to initiate intensive care treatment should, according to perinatal professionals, preferably be made using the shared decision making (SDM) model 95% strongly agreed (Fig. 3). There was less preference for the other models, of all perinatal professionals 27% agreed with the informed and 13% with the paternalistic model as preferred decision-model. There was a significant disagreement within the informed model; obstetricians mainly agreed and neonatologists mainly disagreed with this model.

Current decisions were mostly made by the parents and doctor together (99%). Of those decisions, 28% stated that the professional opinion is decisive, 24% said parents and professional were equally decisive and for 47% the parental opinion was decisive. In these, there were no differences between obstetricians and neonatologists.

#### Other

Six potential indicators of high quality of prenatal counselling were rated. In order of importance, the indicator *health care professional and parents take the decision together equally (shared-decision making)* scored highest (86% of the participants thought this was a fairly good or very good indicator), followed by *when the parents are very satisfied with the consultation* (78% fairly good or very good*)* and *when the content and percentages are medically accurate* (68% fairly good or very good*)*. Lower scores were found for *when the health care professional is very satisfied with the consultation* (44% fairly good or very good*), when all possible complications of premature delivery are discussed* (37% fairly good or very good*)* and *the length of the consultation – the longer, the better/more accurate* (4% fairly good or very good*).*

## Discussion

This nationwide study on prenatal counselling includes both obstetricians and neonatologists from all level III perinatal care centers. In the domain of *organization*, perinatal professionals preferred joint counselling by both the obstetrician and neonatologist, protocols for several aspects of prenatal counselling, supportive material, and the neonatologist to join counselling starting at 23-24 weeks GA. In the domain of *content*, the most important topics to discuss were: mortality, morbidity, intubation/ventilation and intraventricular hemorrhage. Perinatal professionals wanted national or hospital based outcome statistics. In the domain of *decision-making*, perinatal professionals preferred the SDM-model to decide whether or not to initiate treatment. Results of this study can be used when developing a national framework, combined with the results from parental preferences and qualitative explorations.

### Organization of prenatal counselling

Prenatal counselling done together by the neonatologist and obstetrician was preferred just as recommended internationally [1, 2]. Further qualitative research is required to study why this is not usually done in current care, but a hypothesis is that caregivers are simply not simultaneously available at all hours of the day.

The content of the consultation should be documented in both mother’s and infant’s file instead of just in the mother’s file. It is known that records of antenatal consultations were often lacking important information [37]. A technical barrier might be the absence of a medical record for an unborn baby.

A preference for more guidance of prenatal counselling at the limits of viability was reported. In other countries several guidelines and recommendations have been suggested to support professionals performing this difficult task [1–3, 6, 11]. However, disadvantages were mentioned by Janvier [38], who advocates for an approach where doctors should personalize their information and distinguish what specific information parents need. An individual approach and a guideline might not necessarily conflict: a framework on certain aspects of counselling can be of additional value without standardizing prenatal counselling sessions, especially when it’s not too rigid and incorporates solutions to help professionals personalizing the counselling.

Dutch neonatologists wanted to use more supportive material. Grobman found that 60% of parents asked for written material, in contrast to 15% of the physicians who were concerned that clinical conditions could change so rapidly that static resources would not be effective [19]. In 2012, Muthusamy showed in a randomized controlled trial that supplementation of face-to-face verbal counselling with written information improved knowledge and decreased anxiety in women expecting a premature delivery [39]. Guillen and Kakkilaya suggest benefit by the use of a DA [40, 41].

Currently, at a nonviable GA the neonatologist was not considered to take part in counselling in the Netherlands. This in contrast to e.g. California (survey from 1996) and the Pacific Rim (survey from 1999 to 2000) in which at 22 and 23 weeks GA neonatologists were asked to counsel parents [14, 42] The presence of a neonatologist might be helpful, even to explain the rationale of non-active management and to offer comfort care in live-born, immature infants, although a barrier is present since only neonatologists in tertiary centers are trained to counsel these parents.

### Content of prenatal counselling

Many topics were considered important to discuss. However, time might be limited due to an impending delivery and parents will not remember everything when overloaded with information [43]. Therefore, parents’ view on which content should be discussed is essential. From a caregivers perspective, a vast majority preferred to discuss two of the major disabilities (motor and cognitive impairment), but the other two major disabilities (blindness and deafness) were considered less important. We hypothesize that this might be explained by the higher incidence of impaired mental development and cerebral palsy compared to blindness and deafness [44].

Variable morbidity and mortality rates were communicated in prenatal counselling. It is difficult to pinpoint *the* correct percentages for the Dutch situation since during this survey no Dutch outcome data were available, and international statistics vary. A considerable number of neonatologists did not even mention prognostic statistics. Statistics may not always be of additional value to parents. Boss found that physicians’ predictions of morbidity and death are not central to parental decision-making regarding delivery room resuscitation [33]. Janvier rightly appoints the disadvantages of using statistics, i.e. that percentages might not be understood, that its interpretation is framing-dependent and that percentages do not predict the outcome for the individual baby [38]. Nevertheless, Partridge found that *“more data on outcomes”* was recommended for NICU counselling by parents, suggesting that parents want to be informed about prognostic statistics [35].

### Decision-making in prenatal counselling

SDM was the preferred decision-model at the threshold of viability, which is consistent with other studies [2, 4, 11, 34, 35, 45]. Although in current prenatal counselling 99% of the decisions are made by doctor and parent together, 28% of the caregivers state that *their* decision is decisive. It is likely that caregivers might not be fully aware of the way they perform both their current counselling nor that they understand what SDM actually means. SDM is defined as clinicians and patients making decisions together using the best available evidence. This definition states that patients are encouraged to think along and benefits and harms are discussed together [46]. For the implementation of SDM, ready access to evidence based information about treatment options must be met, as well as guidance on how to weigh up the pros and cons of different options and a supportive clinical culture that facilitates patient engagement. Although neonatologists agreed that a DA could be helpful, earlier studies suggested a paternalistic approach [28] and even in this current survey, some of the participants did endorse the informed and/or paternalistic model as well as SDM.

### Other

Participants regarded the implementation of SDM a good indicator for a high quality consultation. Furthermore, they thought an important indicator is *when parents were very satisfied with the consultation* – more important than the satisfaction of the professional. Therefore it is of utmost importance to reveal the preferences of parents in the prenatal counselling. Especially since it is known that views of professionals and parents might differ [47, 48]. Input of professionals and parents should be used for the development of (local) recommendations for prenatal counselling in extreme prematurity.

#### Strengths and limitations

The strongest aspect of this study is its nationwide character, together with an adequate response rate. Part of the survey was directly related to content of the Dutch guideline on perinatal practice, making it relevant for daily practice. This guideline recommends counselling but without giving tools to do so. Our nationwide PreCo study has been set up to examine this counselling, starting with this first exploration of preferred and current counselling.

The limitation of the survey methodology is a potential discrepancy between answers given and actual practice. Besides, direct observations of the counselling conversations could potentially reveal other strengths and weaknesses than we have questioned in this survey, especially interpersonal communication is not easily highlighted in a survey. Due to the inclusion period, effects of experience or learning cannot be ruled out. Furthermore, these Dutch results may not be generalized to an international population. However, both guidelines and a ‘gray zone of viability’ exist worldwide, and although these are not exactly similar to the Dutch counterpart, general conclusions might be applicable.

## Conclusion

This first study on prenatal counselling in the Netherlands revealed differences between preferred and current counselling, and between obstetricians and neonatologists, suggesting a potential for improvement. Further studies looking into the barriers of preferred prenatal counselling [49] could be used to make improvements. Also, preferences of parents will be investigated.

Variation in prenatal counselling is in the best interest of the patient when due to individual (maternal or fetal) characteristics or parental beliefs. When, however, variation is due to unclear background information, insufficient organizational support or incorrect personal habits of healthcare providers, it is not in the best interest of the patient. The use of a nationally developed and supported framework might improve quality of prenatal consultation and even give more scope for individualization.

## Additional files

Additional file 1: Survey neonatologists. Survey presented to the neonatologists, translated from Dutch to English. *Note: The actual survey was sent out online, with a different lay-out.* (PDF 239 kb)

Additional file 2: Survey obstetricians. Survey presented to the obstetricians, translated from Dutch to English. *Note: The actual survey was sent out online, with a different lay-out.* (PDF 232 kb)

## Abbreviations

DA: decision-aid
F.ex: Fisher exact test,
GA: gestational age
MWU: Mann Whitney U test
SDM: shared-decision-making,

## Footnotes

1 When in this manuscript the *perinatal professionals* were mentioned: both obstetricians and neonatologists are meant. Since some in-depth questions were asked only to one of the disciplines, we then noted the applicable discipline (either neonatologists or obstetricians)
