# Correction to: Preferred prenatal counselling at the limits of viability: a survey among Dutch perinatal professionals

**DOI:** 10.1186/s12884-018-1680-x

**Published:** 2018-02-19

**Authors:** Ms Rosa Geurtzen, Arno Van Heijst, Rosella Hermens, Hubertina Scheepers, Mallory Woiski, Jos Draaisma, Marije Hogeveen

**Affiliations:** 1 Amalia Children’s Hospital, Department of Pediatrics, Radboud university Medical Center, PO Box 9101, 6500HB Nijmegen, The Netherlands; 2 Scientific Institute for Quality of Care, Radboud university medical center, Nijmegen, The Netherlands; 3 Department of Gynecology, Maastricht UMC+, Maastricht, The Netherlands; 4 Amalia Children’s Hospital, Department of Gynecology, Radboud University Medical Center, Nijmegen, The Netherlands

## Correction

Following publication of the original article [1], the corresponding authors wrote to say that there was a mistake in the transfer of her article to PubMed: her name is R Geurtzen, but the pdf-file names her as ms R Geurtzen and pubmed names her as MR Geurtzen.

The original article has been updated.
