# Supplementary material for: Preferred prenatal counselling at the limits of viability: a survey among Dutch perinatal professionals
Source: BMC Pregnancy Childbirth. 2018 Jan 3;18:7. doi: 10.1186/s12884-017-1644-6 (PMC5751814; doi:10.1186/s12884-017-1644-6)
Supplement: Supplementary file 2 — Survey obstetricians. Survey presented to the obstetricians, translated from Dutch to English. Note: The actual survey was sent out online, with a different lay-out. (PDF 232 kb) [file 12884_2017_1644_MOESM2_ESM.pdf]

Dear participant,

First, we would like to thank you for participating in our study on prenatal counseling in the case of an expected extremely premature delivery.

Since the introduction in October 2010 of the new guideline "perinatal practice in extreme premature delivery" by the NVOG and NVK, active care can be offered at 24 weeks of gestation, after careful consultation with parents. Regarding decisions for neonates born at 24 weeks of gestation, parental consent is deemed necessary. As consequence of the implementation of this national, multidisciplinary guideline, structured prenatal counseling becomes even more important.

In current practice, prenatal counseling appears to be heterogeneous. There are differences between counseling conversations in content, style and organization. Our pilot study examining prenatal counseling in a simulated setting supports this. Furthermore, international studies confirm these differences. Besides, there is no consensus on who should make the final decision (doctor, parents, shared-decision?).

Adequate counseling is of utmost importance. Therefore, it is necessary to investigate the current situation (how is it done now?). Furthermore, we consider your professional opinion as very important and want to know your preferences (how would you want it?). The purpose of this questionnaire is two-fold. First, to shed light on how prenatal counseling is currently provided, and second, to explore what professionals think constitutes preferred prenatal counseling.

Completing the questionnaire will take about 20-25 minutes. It is possible to complete the questionnaire at a later moment, by clicking in the link in your e-mail again.

Thank you again for your cooperation.

Kind regards

Rosa Geurtzen, MD, resident pediatrics, Radboudumc Amalia Children's hospital
Marije Hogeveen, MD, PhD, neonatologist, Radboudumc Amalia Children's hospital
Jos Draaisma, MD, PhD, pediatrician, Radboudumc Amalia Children's hospital
Arno van Heijst, MD, PhD, neonatologist, head of neonatology department, Radboudumc Amalia Children's hospital

NB This actual survey was sent out online, with a different lay-out. Furthermore, 'skip question logic' was build in online, so that, i.e., participants without children didn't need to fill out the question whether any of the children were premature.

Please fill out the attached questionnaire as completely as possible. You may skip questions that you would prefer not to answer. Some questions will cover a specific scenario (mostly a gestational age of $24^{+0/7}$ to $24^{+6/7}$ weeks); others will ask you to provide a gestational age in a given scenario. Questions will ask about the current way of prenatal counseling and the way you think would be the ideal way to provide counseling.

Sometimes we will use the term “in general”; in that case we are referring to the most common situation.

You will face questions covering the organization of the prenatal counseling, the actual content of the consultation, the style of the conversation, and we will ask you to help us formulate the indicators of good prenatal counseling.

Once again, we would like to thank you for participating in this study.

*** Scenario: gestational age of $24^{+0/7}$ to $24^{+6/7}$ weeks ***

Introduction of a case

The next set of questions covers the consultation that you commonly provide to a pregnant woman facing a possible extremely premature delivery by you as a neonatologist or fellow.

The case is an impending premature delivery at a gestational age of $24^{+0/7}$ to $24^{+6/7}$ weeks with the following characteristics: uncomplicated single pregnancy with an average estimated birth weight, no congenital anomalies, unremarkable social and medical history of both parents, unknown gender of the fetus, full course of corticosteroid has been completed, and the fetal monitor shows a good fetal heart rate with normal variability.

Attention! The left column covers the current situation (i.e. how is it handled now), and the right column covers the ideal situation (i.e. how should it be handled).

	Current situation	*Ideal situation*
1. Who generally conducts the consultation with the parents?	0 Neonatologist or neonatology fellow<br>0 Obstetrician or fellow<br>0 Neonatologist *(or neonatology fellow)* and obstetrician *(or obstetrics fellow)* – jointly<br>0 Neonatologist *(or neonatology fellow)* and obstetrician *(or fellow obstetrics)* – not jointly<br>0 Other	0 Neonatologist or neonatology fellow<br>0 Obstetrician or fellow<br>0 Neonatologist *(or neonatology fellow)* and obstetrician *(or obstetrics fellow)* – jointly<br>0 Neonatologist *(or neonatology fellow)* and obstetrician *(or fellow obstetrics)* – not jointly<br>0 Other

2. Is there a guideline or protocol in your hospital for the different aspects of prenatal counseling mentioned below? *(This is not about the content of this guideline or protocol, just about its existence)*

	Current situation	*Ideal situation*
The gestational age at which you, the obstetrician, shouls ask a neonatologist to provide prenatal counseling to the parents	0 Yes<br>0 No	0 Yes<br>0 No
The professional who conducts the consultation with the parents	0 Yes<br>0 No	0 Yes<br>0 No
The topics that should at least be discussed during prenatal counseling	0 Yes<br>0 No	0 Yes<br>0 No
The minimal gestational age for offering intensive treatment at birth	0 Yes<br>0 No	0 Yes<br>0 No
The gestational age (upper/lower limit) at which the parents' opinion can be decisive in whether or not initiate intensive treatment at birth	0 Yes<br>0 No	0 Yes<br>0 No

3. What topics should be addressed during prenatal counseling?

	Yes; must be discussed	*No need to be discussed*
The chance the baby will die (mortality)	0	0
The change the baby will have disabilities (morbidity)	0	0
Various complications of prematurity on different organ systems	0	0
Practical issues regarding delivery and hospitalization	0	0
Obstetrical interventions in fetal distress and the potential maternal complications	0	0

	Mortality rate for the unborn fetus	*Mortality rate for live-born infant*	*Survival rate **without** severe disabilities (when the infant is live-born)*
4. What percentages should be provided during prenatal counseling in the mentioned case?	0 Don't need to be provided<br>0 I don't know<br>0 0-5%<br>0 6-15%<br>0 16-25%<br>0 26-35%<br>0 36-45%<br>0 46-55%<br>0 56-65%<br>0 66-75%<br>0 76-85%<br>0 86-95%<br>0 96-100%	0 I do not provide a percentage<br>0 I do not provide counseling at this gestation<br>0 0-5%<br>0 6-15%<br>0 16-25%<br>0 26-35%<br>0 36-45%<br>0 46-55%<br>0 56-65%<br>0 66-75%<br>0 76-85%<br>0 86-95%<br>0 96-100%	0 I do not provide a percentage<br>0 I do not provide counseling at this gestation<br>0 0-5%<br>0 6-15%<br>0 16-25%<br>0 26-35%<br>0 36-45%<br>0 46-55%<br>0 56-65%<br>0 66-75%<br>0 76-85%<br>0 86-95%<br>0 96-100%

5. What should be used as a source for rates on mortality and morbidity?

0 International figures
0 European figures
0 National figures
0 Hospital-specific figures
0 I do not provide figures

6. Different ways of explaining chances on mortality and morbidity exist, such as positive and negative framing. What should be used during the counseling consultation: positive framing (e.g. the chances for survival) or negative framing (e.g. the chances for dying)?

0 Positive framing
0 Negative framing
0 Both in one consultation
0 It doesn't matter

7. Should supportive material to support the consultation be used ideally?
0 No, not necessary
0 Yes, written information like a flyer
0 Yes; Online information like a website
0 Yes; a decision aid (visual format supporting parents)
0 Yes; a video
0 Yes; something else, i.e. …………….

8. Who decides whether or not to initiate intensive treatment when the baby is born?
0 The medical professional only
0 The professional and parents together, the professional's opinion is decisive
0 The professional and parents together, both opinions are equally decisive
0 The professional and parents together, the parents' opinion is decisive
0 The parents only

9. The following statements refer to the decision making process in the above mentioned case. To what extent do you agree with these statements?

	Strongly agree	*Agree*	*Neither*	*Disagree*	*Strongly disagree*
The decision to initiate intensive treatment at birth should only be made by a health care professional (paternalistic model).	0	0	0	0	0
The decision to initiate intensive treatment at birth should be made by the parents, after prenatal counseling (informed model)	0	0	0	0	0
The decision to initiate intensive treatment at birth should be made by the health care professional and parents together (shared-decision model)	0	0	0	0	0

The following questions are cover the same case as the questions so far. We will repeat the case again.

Scenario: gestational age of $24^{+0/7}$ to $24^{+6/7}$ weeks

The case is an impending premature delivery at a gestational age of $24^{+0/7}$ to $24^{+6/7}$ weeks with the following characteristics: uncomplicated single pregnancy with an average estimated birth weight, no congenital anomalies, unremarkable social and medical history of both parents, unknown gender of the fetus, full course of corticosteroid has been completed, and the fetal monitor shows a good fetal heart rate with normal variability.

10. How would the situations mentioned below alter your possible recommendation towards parents for initiating intensive treatment in the above mentioned case?

	Less likely to advise intensive treatment	*No influence*	*More likely to advise intensive treatment*
A 5-minute Apgar score below 5	0	0	0
Male gender	0	0	0
No corticosteroids administered	0	0	0
Multiple pregnancy	0	0	0
Small for gestational age	0	0	0
Congenital disorders	0	0	0
Pregnancy conceived by IVF or artificial insemination	0	0	0

11. What of the following do you think would be indicators a high quality prenatal consultation?

	Very bad indicator	*Fairly bad indicator*	*Neutral*	*Fairly good indicator*	*Very good indicator*
When the parents are very satisfied with the consultation	0	0	0	0	0
When the health care professional is very satisfied with the consultation	0	0	0	0	0
When the content and percentages are medically accurate	0	0	0	0	0
When all possible complications of premature delivery are discussed	0	0	0	0	0
The length of the consultation – the longer, the better/more accurate	0	0	0	0	0
Health care professional and parents take the decision together equally (shared-decision making)	0	0	0	0	0

Would you like to add any indicators?
………………………………………………………………………………………………

* Questions broken down by gestational age *

Introduction of a new scenario

The next set of questions are broken down based on gestational age, since questions might differ depending on the gestational age.

It is about an impending delivery with variable gestational ages and the following characteristics: uncomplicated single pregnancy with an average estimated birth weight, no congenital anomalies, unremarkable social and medical history of both parents, unknown gender of the fetus, full course of corticosteroid has been completed, and the fetal monitor shows a good fetal heart rate with normal variability.

12. How often do you (as obstetricians) ask for the neonatologist to provide prenatal counseling to the parents at this GA in threatened preterm delivery?

Attention! The left column covers the current situation (i.e. how is it handled now), and the right column covers the ideal situation (i.e. how should it be handled).

	Current situation	*Ideal situation*
22+0 – 22+6 weeks	0 Always (>95%)	0 Always (>95%)
	0 Often (61-95%)	0 Often (61-95%)
	0 Regularly (21-60%)	0 Regularly (21-60%)
	0 Sometimes (5-20%)	0 Sometimes (5-20%)
	0 Never (<5%)	0 Never (<5%)
23+0 – 23+6 weeks	0 Always (>95%)	0 Always (>95%)
	0 Often (61-95%)	0 Often (61-95%)
	0 Regularly (21-60%)	0 Regularly (21-60%)
	0 Sometimes (5-20%)	0 Sometimes (5-20%)
	0 Never (<5%)	0 Never (<5%)
24+0 – 24+6 weeks	0 Always (>95%)	0 Always (>95%)
	0 Often (61-95%)	0 Often (61-95%)
	0 Regularly (21-60%)	0 Regularly (21-60%)
	0 Sometimes (5-20%)	0 Sometimes (5-20%)
	0 Never (<5%)	0 Never (<5%)
25+0 – 25+6 weeks	0 Always (>95%)	0 Always (>95%)
	0 Often (61-95%)	0 Often (61-95%)
	0 Regularly (21-60%)	0 Regularly (21-60%)
	0 Sometimes (5-20%)	0 Sometimes (5-20%)
	0 Never (<5%)	0 Never (<5%)
26+0 – 26+6 weeks	0 Always (>95%)	0 Always (>95%)
	0 Often (61-95%)	0 Often (61-95%)
	0 Regularly (21-60%)	0 Regularly (21-60%)
	0 Sometimes (5-20%)	0 Sometimes (5-20%)
	0 Never (<5%)	0 Never (<5%)
27+0 – 27+6 weeks	0 Always (>95%)	0 Always (>95%)
	0 Often (61-95%)	0 Often (61-95%)
	0 Regularly (21-60%)	0 Regularly (21-60%)
	0 Sometimes (5-20%)	0 Sometimes (5-20%)
	0 Never (<5%)	0 Never (<5%)

13. A pregnant woman as described in the case above is about to deliver at a fetus of the following gestational ages. What would you recommend at each gestation?

	To provide comfort care, intensive care not offered	*To provide comfort care, intensive care possible on parental request*	*Neutral*	*To provide intensive care, comfort care possible on parental request*	*To provide intensive care, comfort care not offered*
22+0 – 22+6 weeks	0	0	0	0	0
23+0 – 23+6 weeks	0	0	0	0	0
24+0 – 24+6 weeks	0	0	0	0	0
25+0 – 25+6 weeks	0	0	0	0	0
26+0 – 26+6 weeks	0	0	0	0	0
27+0 – 27+6 weeks	0	0	0	0	0

14. This question consists of various interventions that could be taken around a possible premature delivery. What would be your personal lower limit for this intervention?

	GA ≥ 22+0 weeks	*GA ≥ 23+0 weeks*	*GA ≥ 23+5 weeks*	*GA ≥ 24+0 weeks*	*GA ≥ 25+0 weeks*	*GA ≥ 26+0 weeks*
Transfer a pregnant woman with an imminent premature delivery into a hospital with a level III NICU.	0	0	0	0	0	0
Administer antenatal corticosteroids	0	0	0	0	0	0
Use of fetal monitor	0	0	0	0	0	0
Perform a Caesarean section for fetal indication	0	0	0	0	0	0
Have a neonatologist present at the delivery	0	0	0	0	0	0
Intubate after birth when necessary	0	0	0	0	0	0
Provide CPR after birth when necessary	0	0	0	0	0	0
Administer epinephrine after birth when necessary	0	0	0	0	0	0

General information
Thank you for filling out this questionnaire. We appreciate it very much.
Finally, we would like to ask you some (personal) demographic questions. Earlier studies have shown that religion and personal experience with prematurity is relevant to this subject. When you prefer not to fill out these questions, you can skip these. Off course, we will handle this information with much care.

15. What is your gender?
0 Male
0 Female

16. In which hospital do you work?
0 AMC Amsterdam
0 VU Amsterdam
0 Leiden UMC
0 Maastricht UMC
0 UMC Groningen
0 UMC Nijmegen
0 MMC Veldhoven
0 Isala Klinieken Zwolle
0 UMC Utrecht
0 Erasmus MC Rotterdam

17. Are you registered as obstetrician?
0 Yes, obstetrician
0 Yes, fellow obstetrics
0 No, gynecoligst with obstetrics as an area of interest
0 No, other ……

18. How many years of experience do you have in your mentioned profession (question 17)? (*when applicable include fellowshipyears*) ….. years

19. What is your age? ….. years

20. Do you have children?
0 Yes
0 No

21. Were any of your children born at a gestational age lower than 27 weeks?
0 Yes
0 No

22. What is your religious background?

0 Christian (Catholic)
0 Christian (Protestant)
0 Jewish
0 Muslim
0 Other, please specify …
0 No religion
0 I do not want to answer this question

23. How important is your religion for you?

0 Extremely important
0 Important
0 Neutral
0 Unimportant
0 Extremely unimportant
0 Does not apply

24. General comments:

……

……

……

……
